# Use of bitemporal NACA score documentation in prehospital emergency medical services– a retrospective study

**DOI:** 10.1186/s12245-024-00605-5

**Published:** 2024-03-07

**Authors:** Michael Eichinger, Sandro Reiterer, Martin Rief, Michael Eichlseder, Alexander Pichler, Philipp Zoidl, Gerhard Prause

**Affiliations:** 1 Division of Anaesthesiology and Intensive Care Medicine 1, Medical University of Graz, Auenbruggerplatz 5, Graz, A-8036 Austria; 2 Division of Anaesthesiology and Intensive Care Medicine 2, Medical University of Graz, Auenbruggerplatz 5, Graz, A-8036 Austria

**Keywords:** Emergency medical services, Documentation, Classification

## Abstract

**Background:**

The assessment of illness severity in the prehospital setting is essential for guiding appropriate medical interventions. The National Advisory Committee for Aeronautics (NACA) score is a validated tool commonly used for this purpose. However, the potential benefits of using bitemporal documentation of NACA scores to capture the dynamic changes in emergency situations remain uncertain. The objective of this study was to evaluate the potential benefit of bitemporal NACA score documentation in the prehospital setting, specifically in assessing the dynamic changes of emergencies and facilitating quality improvement through enhanced documentation practices.

**Methods:**

In this retrospective study, data from prehospital emergency patients were analyzed who received care from the physician response unit between January 1, 2018, and May 31, 2021. Patient demographics, NACA scores, indications for emergency care, and changes in NACA scores were extracted from medical records. Statistical analyses were performed to examine the associations between NACA scores, emergency categories, indications, and changes in NACA scores.

**Results:**

The study included 4005 patients, predominantly categorized as NACA III (33.7% at initial assessment, 41.8% at subsequent assessment) and NACA IV (31.6% at initial assessment, 22.4% at subsequent assessment). There was a significant improvement in NACA scores during the provision of prehospital care (*p* < 0.01). Notably, prehospital emergencies attributed to internal medical, neurological, traumatic, and paediatric causes demonstrated significant improvements in NACA scores (*p* < 0.01). Gender-specific differences were also observed.

**Conclusion:**

Our study suggests that the bitemporal documentation of NACA scores can be advantageous in the prehospital setting and may have implications for research, practice, and policy.

**Supplementary Information:**

The online version contains supplementary material available at 10.1186/s12245-024-00605-5.

## Key message

1) The NACA score is a validated tool used to categorize illness severity in the prehospital setting. However, the utility of bitemporal NACA score documentation and its impact on understanding the dynamic changes of emergencies remained unknown.

2) This study adds evidence regarding the benefit of bitemporal NACA score documentation in the prehospital setting. It demonstrates that bitemporal assessment provides valuable information on the dynamic changes of emergencies, facilitating a better understanding of the effectiveness of prehospital interventions.

3) The findings of this study may have implications for research, practice, and policy in prehospital emergency care. The use of bitemporal NACA score documentation can enhance the monitoring of treatment effectiveness in the prehospital setting. This study may encourage further research into the implementation and standardization of bitemporal assessment protocols.

**Supplementary Information:**

The online version contains supplementary material available at 10.1186/s12245-024-00605-5.

## Introduction

In the field of prehospital emergency medicine, accurate and timely assessment of illness or injury severity is critical to making appropriate treatment decisions or even triage in case of multiple patients. The National Advisory Committee for Aeronautics (NACA) score is a commonly used tool for this purpose. Developed in the late 1960s, it was one of the first practical methods for assessing the severity of injuries in patients. Originally, its development was in collaboration with the National Advisory Committee for Aeronautics and the German Aerospace Centre to assess the health of astronauts [1, 2].

Initially, it categorized injuries into seven severity grades based on specific injury groups and was determined 24 h after hospital admission. In 1980, Tryba et al. modified the NACA score, providing a general clinical definition for the seven grades and setting the patient classification time at the end of the emergency operation. This modified index and classification for internal emergencies are now widely used in German-speaking emergency services for pre-clinical severity grading of trauma patients [2, 3].

The NACA score’s grades are determined primarily based on the clinical impression of the patient’s condition in the presence of life-threatening illness or injury, graded on a scale of 0 to VII (Table S1). A score of 0 indicates no apparent illness or injury, while a score of VII represents a deceased patient. These grades reflect the immediate life threat an acute condition or injury poses. The grading allows the same injury to be classified into different severity levels based on accompanying circumstances, which can lead to a wide variation in the Injury Severity Score (ISS) for a given NACA grade [2]. The NACA score correlates well with expected morbidity and mortality, making it useful for demographic description in emergency medical systems and retrospective analysis of the need for emergency medical services (EMS) and transport methods at the scene of an incident. However, for a more differentiated prehospital assessment based on physiological parameters, the NACA score should be supplemented by a physiologically based prehospital severity score [2].

Therefore, the NACA score is a straightforward and easy-to-use scoring system that enables EMS providers to rapidly evaluate a patient’s condition and analyse a case-mix for qualitative assessments of the service and audits. Even for the evaluation of triage decisions, the NACA score was used to assess its usefulness [4].

The NACA score’s simplicity is one of its primary advantages, making it ideal for use in high-pressure, time-sensitive situations. Additionally, it serves as a reliable means of communicating a patient’s condition to other healthcare providers, including emergency department staff or physicians where this information is handed over to and classifies patients to receive specific bundles of care [5].

While the NACA score is quick to assess, it is also subjective and reliant on the experience of the healthcare provider performing the evaluation. Moreover, studies have shown that the NACA score has strong predictive value for patient outcomes, with higher scores associated with a greater likelihood of hospital admission, longer hospital stays, and higher mortality rates [6–8].

In Austria, the NACA score is documented uniformly throughout physician-staffed emergency medical services. Multiple NACA score assessments can be beneficial in tracking changes in a patient’s condition and guiding treatment decisions in the prehospital setting, according to Alessandrini et al.‘s prospective study [5].

Another letter by Dami et al. concluded that the NACA score can be a valuable predictor of clinical outcomes in the prehospital and hospital settings [7].

While the NACA score is a useful tool in the prehospital setting, it is only typically recorded once, which can make it challenging to track changes in a patient’s condition over time. A simple example highlighting this would be if a prehospital care team is tasked to an unconscious patient suffering from hypoglycaemia. Initially, this is a life-threatening (NACA V) situation that can easily be solved by experienced teams that might even leave the patient at home if this is considered safe (NACA 0). One-time assessment of this emergency via the NACA score could underestimate the dynamic of the situation and might lead to wrong assumptions regarding this precious resource of prehospital care teams.

To address this our prehospital physician response unit (PRU) documents the NACA score twice during each mission. This documentation approach allows for more precise tracking of changes in the patient’s condition over time and provides valuable information for feedbacking treatment decisions.

To evaluate the feasibility and usefulness of documenting the NACA score twice and the impact of the dynamic changes of an emergency, we conducted a study at our PRU. Our hypothesis was that the bi-temporal assessed NACA score uncovers positive changes in most missions.

## Methods

### Study setting and population

We conducted a retrospective analysis of prehospital emergency patients who were assessed by our PRU team at the University Hospital in Graz, Austria, from 1st January 2018 until 31st May 2021. This specific PRU operates mainly in urban areas and is staffed by a prehospital care doctor and a paramedic. Multiple diagnostic and therapeutic actions can be used, including blood gas analysis, ultrasound, invasive procedures like arterial lines, thoracostomies, resuscitative endovascular balloon occlusion of the aorta (REBOA) up to thoracotomies. Even though this PRU might be specifically equipped compared to others, the general treatment options are the same as most of the other PRUs in Austria and Germany. The PRU is usually staffed with consultant-grade physicians experienced in prehospital care. However, a variety of anaesthetists, intensivists, and surgeons work there. We included all patients with complete documentation of the twice assessed NACA-Scores. We excluded patients who did not fulfil this criterion, as well as patients who received an initial NACA score of VI or VII, were not transported at all, or had implausible task times. The reason for excluding patients who required cardiopulmonary resuscitation (CPR) – NACA VI - upon the physician’s arrival from the primary analysis was that the improvement of this situation is highly dependent on various factors (like delay, lay-CPR, causes of the cardiac arrest, etc.), that we were unable to assess with the retrospective study design. The SQUIRE 2.0 reporting guideline was used to compile the manuscript.

### Key outcome measures

The primary aim of this study was to evaluate the feasibility through a potential benefit of bitemporal NACA (first (NACA 1st) and second (NACA 2nd) assessments) score documentation in the prehospital setting, specifically in assessing the dynamic changes of emergencies and facilitating quality improvement through enhanced documentation practices. ‘NACA 1st’ was defined as the NACA score assessed at the beginning of a mission, and ‘NACA 2nd’ was assessed before hand-over at the end of the same mission. To avoid selection bias, we stratified patients based on their dominant underlying health problem, such as trauma, internal medical, neurological, paediatric, gynaecological, surgical, or other emergencies. Therefore, patients were not overlapped between groups. The secondary aims were to correlate the duration of prehospital management with the initial NACA score (NACA 1st), regardless of the emergency category, and to estimate gender-specific differences of the initial NACA-score evaluation.

### Data collection and statistical analysis

All necessary data were extracted from the electronic data documentation system of our PRU team. Descriptive statistical analyses were used to assess the primary aim within the same emergency categories. Patients were categorized into three groups based on their NACA score changes during the same emergency (improved, no change, and worsened), and listed with their mean age and percentage in each group. The mean, standard deviation, and mean difference were analysed and compared using the Wilcoxon-signed rank test. McNemar-Bowker test was used to assess the polytomous variables in change of NACA scores within the same PRU operations. For secondary analyses, Spearmann’s rank correlation was utilized for the NACA^1st^/time correlation and the Mann-Whitney U test for gender-specific differences. Global statistical significance level was set at 5%.

IBM® SPSS® Statistics (Version 26) was used for statistical analyses.

## Results

**Fig. 1:**
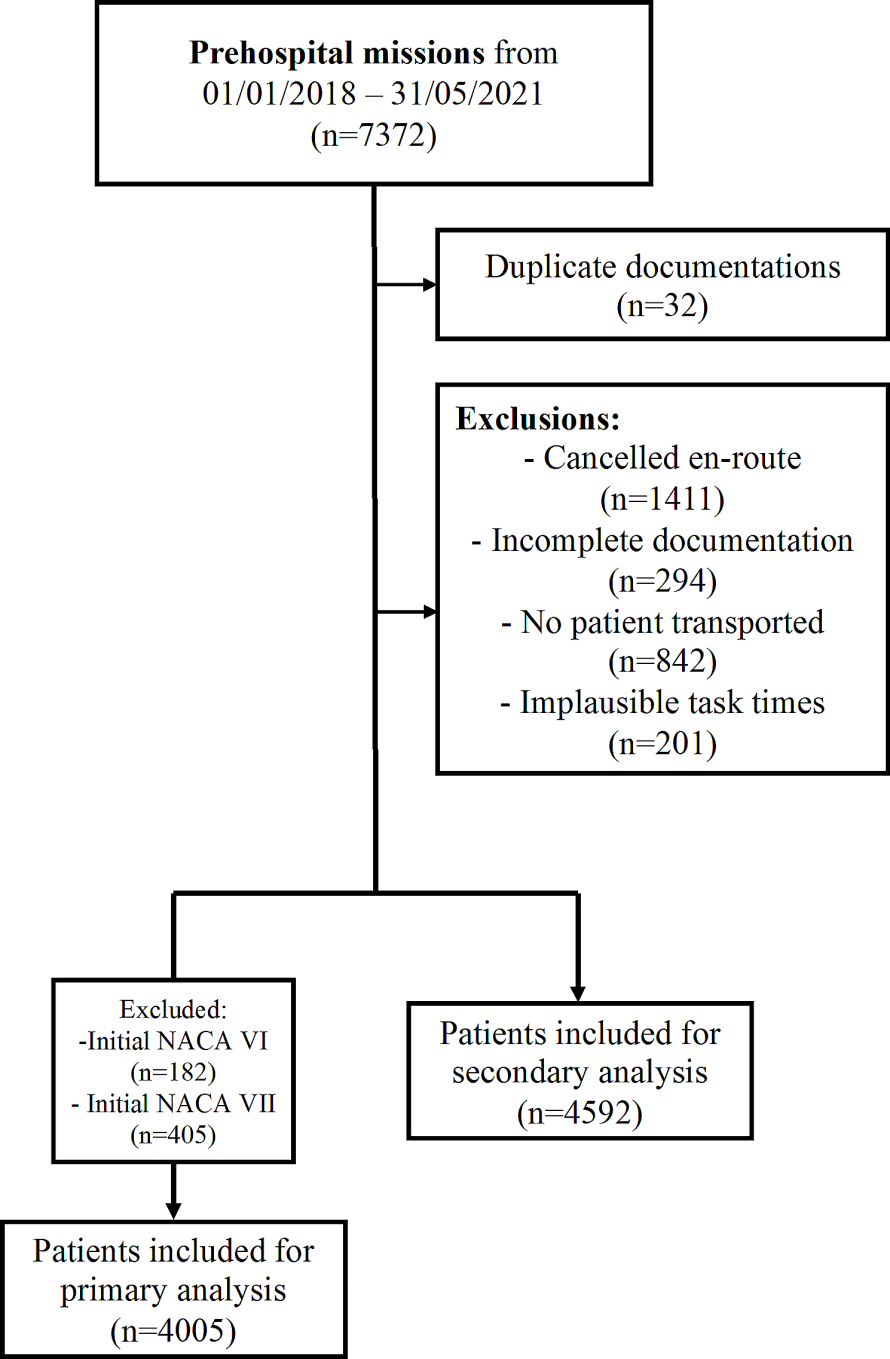
Flowchart. **Legend**: n = number. NACA = National Advisory Committee for Aeronautics Score

The study included a total of 4,592 prehospital emergency patients (Fig. 1). Of these, 51.1% were male, 43.1% female, and 5.7% of unknown gender (Table S2). The majority of the patients were categorized as NACA III at first (moderate to severe emergency), followed by NACA IV (potentially life-threatening) (Table S3). There were very few patients in the NACA I (minimal health problem) category (NACA 1st 0.3% vs. NACA 2nd 0.4%), while the proportion of patients with NACA V (acute life-threatening problem, NACA 1st 10.7% vs. NACA 2nd 8.3%) and NACA VI (cardio-pulmonary resuscitation, NACA 1st 3.9% vs. NACA 2nd 1.3%) categories were higher, respectively (Table S3).

After excluding patients with initial NACA VI and VII, 4,005 patients were available for primary analysis. The 4,005 emergency missions were divided into internal medical, neurological, traumatic, paediatric, gynaecological, surgical, and other causes (Table 1). Table 1 displays the indications for collection of the two NACA scores and the percentage of cases, indicating that internal emergencies (51.2%) were the most common, followed by neurological (16.0%) and traumatic (15.6%) emergencies. The greatest percentage improvement (reduction) in NACA score was observed in emergencies with an internal cause (22.1%), followed by neurological (17.5%) and paediatric problems (17.4%) (Table 2). The NACA scores where no change was observed were fairly evenly distributed between 76.4% (internal emergencies) and 94.1% (gynaecologic emergencies) (Table 1). Surgical emergencies had the highest percentage of worsened NACA scores (3.1%), with the percentage of worsened scores being very low (0-3.1%) (Table 1). Table 2 illustrates these changes, including all NACA score options.

**Table 1:** *Indications and outcomes of bitemporal NACA score assessment*

			NACA score								
	Total		Improved			No change			Worsened		
	n	(%)	n	(%)	Age	n	(%)	Age	n	(%)	Age
Internal	2050	(51.2)	452	(22.1)	66	1566	(76.4)	66	32	(1.6)	72
Neurological	641	(16.0)	112	(17.5)	61	522	(81.4)	63	7	(1.1)	61
Trauma	624	(15.6)	81	(13.0)	50	540	(86.5)	54	3	(0.5)	56
Paediatric	453	(11.3)	79	(17.4)	6	372	(82.1)	7	2	(0.4)	2
Gynaecologic	51	(1.3)	3	(5.9)	32	48	(94.1)	30	0	(0.0)	-
Surgical	32	(0.8)	3	(9.4)	28	28	(87.5)	64	1	(3.1)	70
Others	154	(3.9)	23	(14.9)	58	128	(83.1)	54	3	(2.0)	62
Total	4005	(100.0)	753	(18.8)	56.7	3204	(80.0)	55.6	48	(1.2)	65.8

**Legend**: n = number. NACA = National Advisory Committee for Aeronautics Score. Frequencies and indications of prehospital missions with respect to twice-assessed NACA score outcome. Age in years.

**Table 2:** *Illustration of transformation between NACA 1st and NACA 2nd *

		NACA 2^nd^							Total
		I	II	III	IV	V	VI	VII	
NACA 1st	I	12	3	0	0	0	0	0	15
	II	4	493	6	0	0	0	0	503
	III	1	101	1435	9	1	0	0	1547
	IV	1	60	453	917	15	4	0	1450
	V	0	4	27	102	347	9	1	490
	VI	0	0	0	1	19	47	113	180
	VII	0	0	0	0	0	0	232	232
Total		18	661	1921	1029	382	60	346	4417

**Legend**: NACA = National Advisory Committee for Aeronautics Score. Calculated with McNemar-Bowker test. Dark grey fields display worsened (higher) NACA scores, the diagonal numbers display no change, light grey display improved (lower) NACA scores within the same mission. Patients with missing NACA 2nd were excluded, hence the different number of included patients.

The Wilcoxon signed-rank test revealed significant improvements in mean NACA scores in internal emergencies (NACA 1st 3.58 to NACA 2nd 3.35), neurological (NACA 1st 3.52 to NACA 2nd 3.34), trauma (NACA 1st 3.24 to NACA 2nd 3.1), paediatric (NACA 1st 3.17 to NACA 2nd 2.98) emergencies, and emergencies with an undefined cause (NACA 1st 3.85 to NACA 2nd 3.68) (*p* < 0.01 for all mentioned tests) (Table 3).

**Table 3:** *Association of indications and NACA score*

	n (%)*	Mean NACA 1st score (σ)	Mean NACA 2nd score (σ)	Mean diff	p-value
Internal	2050 (51.2)	3.58 (0.890)	3.35 (0.906)	-0.23	< 0.01
Neurological	641 (16.0)	3.52 (0.870)	3.34 (0.877)	-0.18	< 0.01
Trauma	624 (15.6)	3.24 (0.770)	3.10 (0.729)	-0.14	< 0.01
Paediatric	453 (11.3)	3.17 (0.822)	2.98 (0.745)	-0.19	< 0.01
Others	154 (3.9)	3.85 (0.899)	3.68 (0.968)	-0.17	< 0.01
Gynaecologic	51 (1.3)	3.04 (0.662)	2.98 (0.616)	-0.06	0.08
Surgical	32 (0.8)	3.47 (0.761)	3.44 (0.878)	-0.03	0.70
Total	**4005 (100.0)**	**3.47**	**3.28**	**-0.19**	**< 0.01**

**Legend**: n = number. NACA = National Advisory Committee for Aeronautics Score. Associations of NACA and frequencies and different indications of prehospital missions. p values calculated with Wilcoxon signed rank test. Level of significance *p* < 0.05. *Numbers as absolute values and percentages in brackets.

For the first secondary objective, Spearman’s rho was utilised to determine the correlation between NACA 1st and time, demonstrating a weak positive correlation (*r* = 0.09, *p* < 0.01).

Gender-specific differences associated with the initial NACA score were investigated (refer to Figure S1). Women were evaluated with lower NACA 1st scores (mean 3.75) compared to their male counterparts (mean 3.95) (*p* < 0.01).

## Discussion

The current study indicates the effectiveness of improving NACA scores in most emergency categories through potential meaningful interventions as described by Wilson et al. [9], highlighting the importance of using a standardized scoring system to assess the severity of illness and injury in prehospital emergency patients. Our study supports the reliability of the NACA score described by Weiss et al. [10] and emphasizes its utility in describing the dynamics of an emergency with a bitemporal assessment of NACA score. Notably, we observed a high proportion of patients with moderate health problem levels (NACA III and IV), underlining the need for timely and appropriate prehospital care. Our results show a significant improvement in bitemporal NACA scores among prehospital emergency patients, especially those with internal, neurological, traumatological, and paediatric causes. It is noteworthy that internal, paediatric, and neurological emergencies exhibited the highest rates of improvement in the NACA score. Conversely, surgical emergencies were found to have the highest incidence of worsened NACA scores, although this was a small percentage for all emergencies (up to 3.1%). This observation may be attributed to the fact that certain surgical emergencies, particularly uncontrollable bleeding, cannot be effectively treated in a prehospital setting.

The dynamic changes between the two NACA scores collected in each case showed a high degree of unchanged NACA scores in the NACA II and III categories and a high degree of improvements in NACA scores within a mission in the NACA IV and V categories. This finding suggests good results in terms of reducing NACA scores, especially for patients with a severe health problem. In missions primarily classified as NACA VI, degradation occurred in more than half of the cases. The use of standardized scoring systems, such as bitemporal NACA, can contribute to quality improvement initiatives by providing valuable data for analysis. Furthermore, it can facilitate the tracking of patient progress and evaluation of interventions. Clinicians can identify areas for improvement in prehospital emergency care by evaluating changes in NACA scores over time and develop effective strategies to address these issues. Additionally, the use of standardized scoring systems can promote effective communication and coordination between prehospital and hospital providers, resulting in improved patient care throughout the continuum of care.

Our study also revealed that higher NACA scores were associated with longer on-scene times. Although this finding may seem obvious, it is still interesting because an initial NACA VII score suggests a prolonged scene time without adding value for the patient. However, during such missions, the limited resource ‘PRU’ is blocked for other patients that might benefit from its input. This finding combined with the majority of low NACA missions suggests that prehospital dispatch could be improved with NACA scores as potential markers. This result is supported with the study of Schneider et al. who found that the initial NACA score is indicative for physician’s subjective workload during prehospital emergency care [11].

Moreover, we found gender-specific differences in the initial NACA evaluations of prehospital emergency patients although the distribution between the two sexes was relatively balanced. This finding is consistent with several studies suggesting differences in the evaluation of critical care patients between male and female patients [12, 13]. Further research is needed to investigate the potential reasons for these gender differences in detail.

While a bitemporal NACA documentation system has potential benefits, implementing this system may pose challenges. The additional time and resources required to document NACA scores before and after treatment may prove burdensome for prehospital providers. Variations in the interpretation and application of NACA scoring among providers may also necessitate ongoing training and education to ensure accurate and consistent use of the system.

### Limitations

Our study has several limitations that should be taken into consideration when interpreting the results. Firstly, the retrospective design of the study and reliance on data collected from medical records may introduce the possibility of bias and incomplete documentation. This is particularly relevant in the prehospital setting where documentation can be challenging due to the high-pressure environment. To mitigate this limitation, we excluded prehospital interventions from our analysis, as this was documented too variably. Another limitation of our study is related to the electronic documentation system used, which included a ‘same as on arrival’ button that was implemented to simplify documentation. This may have led to an overestimation of the number of missions that did not change the NACA score, potentially masking even small improvements or declines in patient condition. Nevertheless, this reflects daily practice in the prehospital setting and underscores the need for continued improvement in documentation systems to accurately capture changes in patient condition over time.

On the other hand, the NACA score, by its nature, involves a degree of subjective judgment by the clinician. This subjectivity could lead to an unintentional overestimation of the treatment’s effectiveness, especially in cases where clear, objective improvement measures are not readily available.

## Conclusion

In summary, this study confirms the feasibility and highlights the potential benefits of implementing a bitemporal NACA documentation system and its documentary value in prehospital emergency medicine to optimize quality. The standardized NACA scoring system can help clinicians assess the severity of illness and injury and track patient progress through a more appropriate illustration of a patients’ change of state. Moreover, the use of NACA can facilitate quality improvement initiatives and improve communication and coordination among providers, thereby ensuring that patients receive appropriate care throughout the continuum of care. Although challenges may arise during the implementation of a bitemporal NACA documentation system, the potential benefits justify further exploration and consideration. Overall, this study emphasizes the importance of multiple evaluation of standardized scoring systems in prehospital emergency medicine and the need for ongoing efforts to improve patient outcomes, including potential gender-specific differences in assessment, which should be further explored in future research.

### Electronic supplementary material

Below is the link to the electronic supplementary material.

Supplementary Material 1

## Data Availability

The datasets generated during and/or analyzed during the current study are available from the corresponding author on reasonable request.

## Declarations

### Patient and public involvement

Patients/Public were not involved in this research project due to the retrospective design.
