# Supplementary material for: Use of bitemporal NACA score documentation in prehospital emergency medical services– a retrospective study
Source: Int J Emerg Med. 2024 Mar 7;17:36. doi: 10.1186/s12245-024-00605-5 (PMC10918989; doi:10.1186/s12245-024-00605-5)
Supplement: Supplementary file 1 — Supplementary Material 1 [file 12245_2024_605_MOESM1_ESM.docx]

**Table S1***: NACA-Score classifications.*

| NACA 0 | *No injury or acute medical problem* |
| --- | --- |
| NACA I | *Minimal health problem, no prehospital intervention required* |
| NACA II | *Mild health problem requiring assessment at the Emergency Department* |
| NACA III | *Moderate to severe emergency, not life-threatening* |
| NACA IV | *Severe health problem, potentially life-threatening* |
| NACA V | *Acute, life-threatening problem* |
| NACA VI | *Cardio-pulmonary reanimation (CPR)* |
| NACA VII | *Death* |

**Legend:** NACA= National Advisory Committee for Aeronautics Score.

**Table S2***: Gender related distribution.*

| Total | 4592 |
| --- | --- |
| Male | 2348 (51.1) |
| Female | 1980 (43.1) |
| Unknown | 264 (5.7) |

**Legend:** Gender distribution of included patients (including NACA VI+VII). Numbers displayed as absolute values and percentages in brackets.

**Table S3***: Distribution of NACA 1^st^ and NACA 2^nd^.*

|  | | **NACA 1^st^** | **NACA 2^nd^** |
| --- | --- | --- | --- |
|  | **1** | 15 (0.3) | 18 (0.4) |
|  | **2** | 503 (11.0) | 661 (14.4) |
|  | **3** | 1547 (33.7) | 1921 (41.8) |
|  | **4** | 1450 (31.6) | 1029 (22.4) |
|  | **5** | 490 (10.7) | 382 (8.3) |
|  | **6** | 181 (3.9) | 60 (1.3) |
|  | **7** | 403 (8.8) | 346 (7.5) |
|  | **Total** | 4589 (99.9) | 4417 (96.2) |
| **Missing data** |  | 3 (0.1) | 175 (3.8) |
| **Total** | | 4592 (100.0) | 4592 (100.0) |

**Legend:** NACA= National Advisory Committee for Aeronautics Score. Illustration of total missions specified with a bitemporal NACA score and missing data. Numbers displayed as absolute values and percentages in brackets.

**Figure S1**: *Gender differences of NACA 1^st^.*


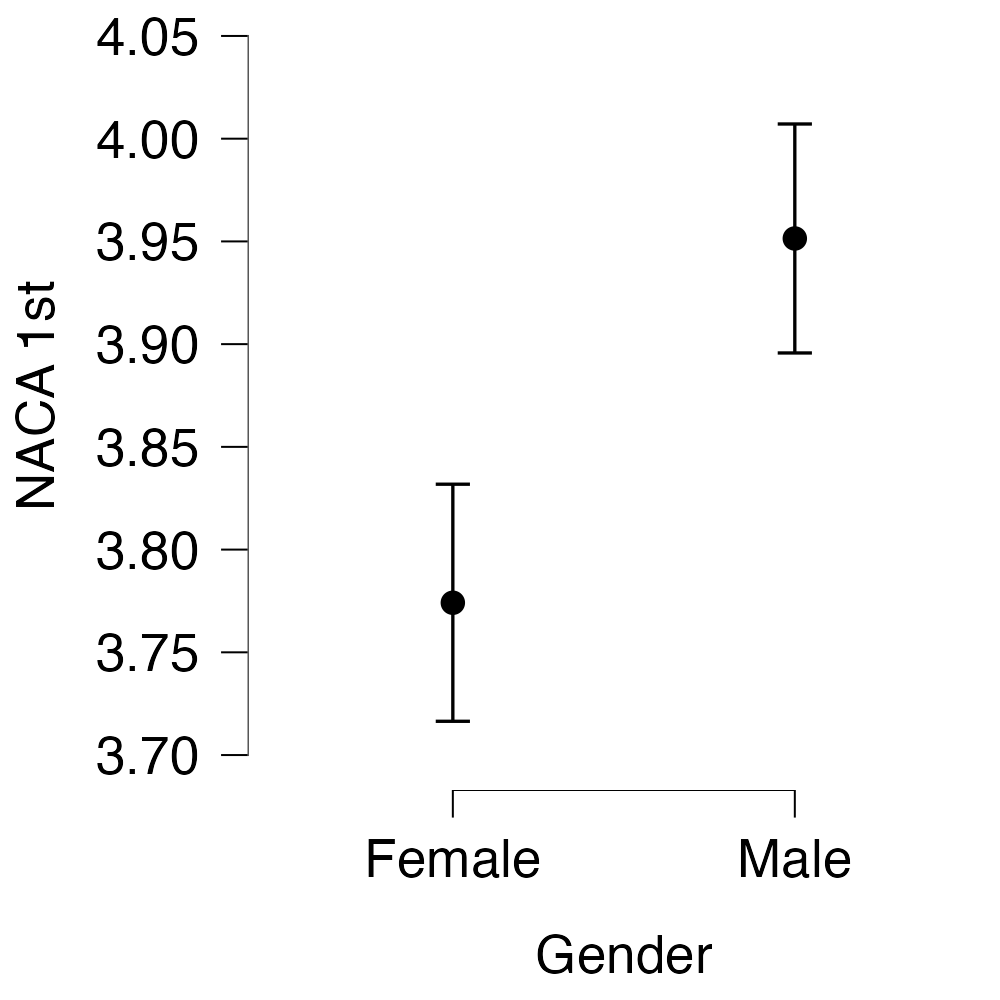


**Legend:** NACA= National Advisory Committee for Aeronautics Score. Illustration of interval plots of initial NACA score (NACA 1^st^) compared between female and male patients.
